# Robot-assisted partial nephrectomy in patients aged 75 years or older – comparing the risk of complications with their younger counterparts

**DOI:** 10.1007/s40520-024-02751-5

**Published:** 2024-05-08

**Authors:** Rasmus D. Petersson, Mikkel Fode, Malene H. Niebuhr, Badal S. Rashu, Frederik F. Thomsen

**Affiliations:** 1 Department of Urology, Copenhagen University Hospital, Herlev and Gentofte Hospital, Herlev, Denmark; 2 Department of Urology, Zealand University Hospital, Roskilde, Denmark

**Keywords:** Renal cell carcinoma, Robot assisted, Partial nephrectomy, Complication, Age, Geriatric surgery

## Abstract

**Background & aim:**

More elderly patients are diagnosed with kidney tumors where partial nephrectomy is technically possible. We investigated whether patients ≥ 75 years old had an increased risk of complications following robot-assisted partial nephrectomy (RAPN) compared to younger patients.

**Methods:**

Retrospective, consecutive study including patients who underwent RAPN between May 2016 – April 2023. Preoperative data, operative data and complications within 90 days were recorded by patient record review. Complications were classified according to Clavien-Dindo (CD).

**Results:**

451 patients underwent RAPN and a postoperative complication was recorded in 131 (29%) patients of which 28 (6%) were CD ≥ III. Any postoperative complication was recorded in 24/113 patients (21%) < 55 years, 40/127 patients (31%) 55–64 years, 45/151 patients (42%) 65–74 years, and 22/60 patients (37%) ≥ 75 years. Comparable numbers for a CD ≥ III postoperative complication were 2/113 (2%) < 55 years, 6/127 (7%) 55–64 years, 12/151 (8%) 65–74 years, and 5/60 (8%) ≥ 75 years. In multivariate logistic regression analysis, patients ≥ 75 years had a non-significant increased risk of complications when controlling for preoperative variables (OR 1.82 [95% CI 0.80–4.13]) or perioperative variables (OR 1.98 [95% CI 0.86–4.58]) compared to patients < 55 years. Two patients died postoperatively. Both were ≥ 75 years (2/60, 3%).

**Discussion and conclusions:**

Selected patients ≥ 75 years can undergo RAPN without a significantly increased risk of postoperative complications. However, a mortality rate of 3% in this age group indicates that these patients are frail when postoperative complications occur.

**Supplementary Information:**

The online version contains supplementary material available at 10.1007/s40520-024-02751-5.

## Introduction

Renal cell carcinoma (RCC) represents approximately 3% of all cancers [1]. In the European Union alone, 100 000 patients are diagnosed with RCC each year. The risk of RCC increases with age [2], and the incidence has been increasing over the last 20 years and is projected to continue to increase in the future [3]. Most of this increase is caused by incidental findings of small renal masses [4–7]. Moreover, people worldwide are living longer, and the proportion of older persons in the population is increasing [8]. Taken together more elderly patients will be diagnosed with localized RCC.

The preferred curative treatment modality for localized RCC is partial nephrectomy, when technically feasible, since it better preserves renal function compared to radical nephrectomy while still providing excellent oncological outcome [3, 9, 10]. However, there are caveats, as surgical treatment is not without risk, and many patients are diagnosed with small tumors that will pose little threat for many years [11, 12].

Previous studies have shown that frail patients are at an increased risk of postoperative complications after surgery [13]. Treatment selection for elderly patients is influenced by clinician bias, patient preferences and limited data on surgery within this age group [14, 15]. This highlights the need for knowledge about the effects of robot-assisted partial nephrectomy (RAPN) in this patient group to aid treatment selection. The purpose of this study was to investigate if patients aged 75 years or older are at an increased risk of suffering postoperative complications after RAPN compared to younger patients.

## Material & methods

Retrospective, consecutive study including patients who underwent RAPN for nonmetastatic, localized renal tumors between May 2016 – April 2023 at the Department of Urology, Copenhagen University Hospital, Herlev and Gentofte Hospital, Herlev, Denmark. The following data were recorded by electronic patient record review: preoperative data (age, gender, Charlson Comorbidity Index [CCI] [16], American Society of Anesthesiologists classification [ASA] [17], body mass index [BMI], smoking status, kidney function, tumor size), operative data (duration of the procedure, warm ischemia time, blood loss), and complications within 90 days. In order to minimize bias, data retrieval and analysis was not performed by the primary operating surgeons (data retrieval and analysis was performed by R.D.P and F.F.T). Postoperative complications were classified according to the Clavien-Dindo classification (CD) [18]. The study received ethical and legal approval from the regional center for register research of the Capital Region of Denmark according to Danish law (journal number: R-23018007).

Descriptive statistics were used, and the chi-square test was used to test differences in baseline characteristics between age groups. We stratified in age groups (< 55 years, 55–64 years, 65–74 years and ≥ 75 years) in order to compare the risk of complications in different age categories. Odds ratios (OR) with 95% confidence intervals (CI) for postoperative complications were assessed with uni- and multivariate logistic regression analyses controlling for pre- and perioperative variables. The variables controlled for were, preoperative: gender (female, male), CCI (0, 1, ≥ 2), ASA (1,2, ≥ 3), BMI (< 25,25–29, ≥ 30), smoking status (never, current, former), tumor size (quartiles), and perioperative: duration of the procedure (quartiles), warm ischemia time (quartiles), and blood loss (quartiles). All tests were two-sided, and the significance level was set to *p* < 0.05. Statistical analysis was performed with R version 4.0.0 (R Foundation for Statistical Computing, Vienna, Austria).

## Results

A total of 451 patients underwent RAPN, and no patients were lost to follow-up. Baseline characteristics are presented in Table 1.

**Table 1:** Baseline characteristics of 451 patients who underwent robot assisted partial nephrectomy for localized nonmetastatic kidney tumour stratified on age

			Age				
			< 55	55–64	65–74	≥ 75	
			n(%) = 113(25)	n(%) = 127(28)	n(%) = 151(34)	n(%) = 60(13)	*p*
Gender							0.03
	Female		34 (30)	37 (29)	51 (34)	30 (50)	
	Male		79 (70)	90 (71)	100 (66)	30 (50)	
Charlson comorbidity index							0.001
	0		89 (79)	79 (62)	72 (48)	32 (53)	
	1		20 (18)	33 (26)	46 (30)	18 (30)	
	≥ 2		4 (1)	15 (12)	33 (22)	10 (17)	
ASA classification							< 0.001
	1		34 (30)	20 (16)	13 (9)	2 (3)	
	2		66 (58)	76 (60)	85 (56)	33 (55)	
	≥ 3		13 (12)	31 (24)	53 (35)	25 (42)	
Body mass index							0.01
	< 25		38 (34)	36 (28)	56 (37)	29 (48)	
	25–29		27 (24)	39 (31)	54 (36)	18 (30)	
	≥ 30		42 (37)	46 (36)	36 (24)	10 (17)	
	Missing		6 (5)	6 (5)	5 (3)	3 (5)	
Smoking							0.002
	Never		55 (49)	45 (35)	46 (30)	29 (48)	
	Active smoker		32 (28)	32 (25)	37 (25)	8 (13)	
	Former smoker		23 (20)	48 (38)	64 (42)	20 (33)	
	Missing		3 (3)	2 (2)	4 (3)	3 (15)	
Kidney function, eGFR							< 0.001
	< 75		9 (8)	24 (19)	47 (31)	32 (53)	
	75–89		18 (16)	21 (17)	49 (32)	24 (40)	
	≥ 90		86 (76)	82 (65)	51 (34)	4 (7)	
	Missing		0 (0)	0 (0)	4 (3)	0 (0)	
Former surgery							0.90
	No		76 (67)	90 (71)	107 (71)	43 (72)	
	Yes		37 (33)	37 (29)	44 (29)	17 (28)	
		Open	23 (21)	22 (17)	29 (19)	9 (15)	
		Laparoscopic	14 (12)	15 (12)	15 (10)	8 (13)	
Clinical stage							0.04
	T1a		95 (84)	111 (87)	124 (82)	42 (70)	
	T1b		18 (16)	14 (11)	26 (7)	18 (30)	
	T2a		0 (0)	2 (2)	1 (1)	0 (0)	
Tumor size, mm							
	Median(IQR)		26 (20–36)	26 (20–35)	30 (20–40)	34 (27–43)	*p* < 0.001

*Abbreviations* n, patient number; ASA, American Society of Anaesthesiology; ECOG, Eastern Cooperative Oncology Group; IQR, interquartile range

152 (34%) patients were female and 299 (66%) were male. A total of 60 (13%) patients were aged ≥ 75 years (range 75–87 years), 151 (33%) were 74 − 65 years, 127 (28%) were 55–64 years and 113 (25%) were < 55 years. The median operation time was 173 (IQR 145–204) min, the median warm ischemia time was 15 (IQR 12–19) min, and the median perioperative blood loss was 100 (IQR 50–200) ml. Intraoperatively were 7 (1.5%) procedures converted to radical nephrectomy and 3 (1%) procedures were converted to open surgery, none of the cases were overlapping. There were 8 (2%) intraoperative complications: 4 spleen lesions, 2 diaphragm perforations, 1 kidney vein injury and 1 small bowel perforation. All were managed with either sutures and/or synthetic haemostatic patch. The median length of stay at the hospital was 3 (2–4 IQR) days.

Postoperative complications were recorded in 131 (29%) patients (Table 2).

**Table 2:** Overview of complications stratified on Clavien Dindo score

	Clavien Dindo score					
Complication	I	II	IIIa	IIIb	IVb	V
	n (%)	n (%)	n (%)	n (%)	n (%)	n (%)
Delirious and kidney dysfunction						1 (0.2)
Kidney dysfunction						1 (0.2)
Multiorgan dysfunction					1 (0.2)	
Bleeding	9 (2)	6 (1)		13 (3)		
Uroplania				4 (1)		
Hernia				1 (0.2)		
Abscess			2 (0.4)	1 (0.2)		
Diverticulitis				1 (0.2)		
Infection	1 (0.2)	60, (13)*				
Ileus		4 (1)				
TCI		2 (0.4)				
DVT		1 (0.2)				
Urinary retention	4 (1)					
Other	10 (2)	6 (1)	2 (0.4)	1 (0.2)		
In total	24 (5)	79 (18)	4 (1)	21 (5)	1 (0.2)	2 (0.4)
.						

*42 patients commenced antibiotic solely because of postoperative fever with negative urine and blood culture

*Abbreviations* n, patient number; TCI, transient cerebral ischaemia; DVT, deep vein thrombosis

Complications stratified according to CD grade: 24 (5%) patients had a CD I, 79 (18%) patients had a CD II, and 28 (6%) patients had a CD ≥ III. The most common complication was postoperative infection, which was recorded in 61 (13%) patients; out of these 42/61 (69%) patients commenced antibiotics solely because of postoperative fever, and their subsequent blood and urine cultures were negative, however they were still registered as complications in the analyses. The most frequent major complication (CD ≥ III) was postoperative bleeding, where 13 (3%) patients required intervention with either surgery or embolization.

Two (0.4%) patients died within 90 days of the procedure. Both patients were aged 75 years or older (2/60, 3%), had a CCI of 1, an ASA of 2–3 and were smokers or former smokers. The operative time and perioperative blood loss were in the upper quartile. Both had a prolonged postoperative stay with renal failure (between 13 and 16 days). One died of cardiac arrest while receiving dialysis. The other went into delirium, which could not be reversed.

Complications stratified by age group are presented in Table 3.

**Table 3:** Number of complications stratified on age group and logistic regression analysis of association between age group and postoperative complications

Number of complications				
	Any complication n (%)		CD ≥ III n (%)	
*Age, years*				
< 55	24 (21)		2 (2)	
55–64	40 (31)		9 (7)	
65–74	45 (42)		12 (8)	
≥75	22 (37)		5 (8)	
*Univariate logistic regression*				
	Any complication		CD ≥ III	
	OR	95% CI	OR	95% CI
*Age, years*				
< 55	Ref		Ref	
55–64	1.7	0.95–3.09	4.74	1.21-31
65–74	1.57	0.90–2.81	5.67	1.54-37
≥75	2.15	1.07–4.31	5.05	1.05-36
*Multivariate logistic regression - preoperative variables (Adjusted for: Gender, CCI, ASA, BMI, smoking and tumor size)*				
	Any complication		CD ≥ III	
	OR	95% CI	OR	95% CI
*Age, years*				
< 55	Ref		Ref	
55–64	1.55	0.82-3.00	3.49	0.80–24
65–74	1.35	0.69–2.65	3.78	0.88-26
≥75	1.82	0.80–4.13	3.21	0.57-25
*Multivariate logistic regression - perioperative variables (adjusted for: duration of surgery, warm ischemia time and blood loss)*				
	Any complication		CD ≥ III	
	OR	95% CI	OR	95% CI
*Age, years*				
< 55	Ref		Ref	
55–64	1.29	0.64–2.63	3.2	0.75-22
65–74	1.3	0.65–2.65	2.67	0.62-18
≥75	1.98	0.86–4.58	2.48	0.43-19

*Abbreviations* n, patient number; OR, odds ratio; CI, confidence interval; Ref, reference; CD, Clavien Dindo: CCI, Charlson comorbidity index; ASA, American Society of Anesthesiologists; BMI, Body mass index

In univariate logistic regression analysis, patients ≥ 75 years had a higher risk of a postoperative complication (OR 2.15 [95% CI 1.07–4.31]), including CD ≥ III (OR 5.05 [95% CI 1.05-36]) compared to patients aged < 55 years (Table 3). When controlling for preoperative variables, patients ≥ 75 years had a non-significantly increased risk of any complication (OR 1.82 [95% CI 0.80–4.13]) or CD ≥ III (OR 3.21 [95% CI 0.57-25]) compared to patients < 55 years. Comparable numbers when patients aged ≥ 75 years were compared directly to (1) patients aged 55–64 years: OR 1.48 (95% CI 0.66–3.32) and 1.93 (95% CI 0.43–8.33), and (2) patients aged 65–74 years: OR 1.22 (95% CI 0.59–2.49) and 1.01 (0.26–3.55).

The same was found when controlling for perioperative variables, where patients ≥ 75 years had a non-significantly increased risk of any complication OR 1.98 (95% CI 0.86–4.58) or CD ≥ III OR 2.48 (95% CI 0.43-19) compared to patients < 55 years. Comparable numbers when patients aged ≥ 75 years were compared directly to (1) patients aged 55–64 years: OR 1.45 (95% CI 0.64–3.26) and 0.78 (95% CI 0.18–3.02), and (2) patients aged 65–74 years: OR 1.59 (95% CI 0.72–3.53) and 1.21 (0.28–4.76). A post hoc power calculation showed that the study has a power of 0.78 to show a difference in the risk of any complication between patients aged < 55 years and patients aged ≥ 75 years, with a type I error of 5%.

Large perioperative bleeding (Q4 vs. Q1 OR 2.16 [95% CI 1.11–4.21]) and long warm ischemia time (Q4 vs. Q1 OR 2.28 [95% CI 1.09–4.87]) were the only variables that were significantly associated with an increased risk of any postoperative complications in multivariate analyses. In addition, long operative time (Q4 vs. Q1 OR 4.33 [95% CI 1.02-30]) and large perioperative bleeding (Q4 vs. Q1 OR 3.96 [95% CI 1.06-19]) were the only variables that were significantly associated with an increased risk of CD ≥ III postoperative complications. Full logistic regression models are available in Supplemental Tables 1–2.

## Discussion

In this retrospective consecutive study of 451 patients who underwent RAPN, we found that patients ≥ 75 years had an increased risk of postoperative complications compared to patients < 55 years, but when we controlled for pre- and perioperative variables, older age was no longer significantly associated with an increased risk of complications. Additionally, patients ≥ 75 years had a similar risk of suffering postoperative complications when compared to patients aged 55–64 and 65–74 years. This indicates that age in itself may not be a risk factor for postoperative complications following RAPN. However, with two postoperative deaths in patients ≥ 75 years, it is possible that older patients are frailer when suffering a postsurgical complication.

The main limitation of the study is its retrospective design, which could introduce biases that we were unable to control for as well as underreporting of postoperative complications. Moreover, these data were not suited to identify which patients aged ≥ 75 years were at increased risk of a major complication. The strengths of the study were the complete follow-up and that the data retrieval and analyses were not performed by the primary operating surgeons. Furthermore, postoperative care was similar for the entire cohort, as all patients were treated at the same center.

Our results are in line with those of previous publications [19–25]. In a recent registry study, the risk of a postoperative CD ≥III complication following 1 056 “minimal invasive” partial nephrectomies was 6.2%, and age was not found to be an independent risk factor for a postoperative complication [22]. Sandberg et al. reported comparable complication rates following RAPN in patients aged < 70 years (*n* = 268) vs. ≥70 years (*n* = 71), with 5.6% CD ≥III complications in the elderly cohort [19]. Thomas et al. found that patients aged ≥ 80 years (*n* = 41) did not have an increased risk of postoperative complications following laparoscopic partial nephrectomy compared to patients younger than 80 years (*n* = 791) [23].

The increase in the detection of small renal masses [4, 5] remains a clinical dilemma for physicians and their patients, especially in elderly patients with long expected survival. Some of these masses are benign, which could lead to overtreatment if surgical excision was performed in all these cases [26–29]. Adding a preoperative biopsy to the work-up of small renal masses could improve the diagnostics of benign tumors and thus spare some patients from undergoing unnecessary surgery [30–33]. Additionally, Lane et al. found no difference in RCC-specific or overall survival between patients aged ≥ 75 years diagnosed with cT1 kidney tumors and managed with curative intent (surgery or ablation) vs. observation [34]. On the other hand, older patients are less likely to receive surgical treatment for cancer compared to their younger counterparts, even when adjusting for comorbidity and performance status, and it has been theorized that this could contribute to poorer overall survival [14, 15]. The life expectancy of the average 75-year-old in Denmark is over 10-years. Thus, elderly patients should not be excluded as candidates for curative treatment based solely on their age [35]. This highlights the complexity of treating elderly patients with potential curable RCC and the need for further data to aid decision-making.

Studies on preoperative information which can identify patients at increased risk of postoperative complications are warranted. Also, improving preoperative optimization and postoperative rehabilitation could possibly further increase the benefits of surgery [36]. Finally, there is little information available on patient-related outcomes, such as patient choice and quality of life after cancer treatment, in elderly patients [14].

## Conclusion

Selected patients aged 75 years or older can undergo RAPN without a significantly increased risk of suffering postoperative complications. However, a mortality rate of 3% in this age group indicates that these patients are frail when suffering postoperative complications. Future studies should focus on the optimal selection of surgical candidates as well as improving preoperative optimization and postoperative rehabilitation.

### Electronic supplementary material

Below is the link to the electronic supplementary material.

Supplementary Material 1

Supplementary Material 2

## Data Availability

No datasets were generated or analysed during the current study.
