# Supplementary material for: Robot-assisted partial nephrectomy in patients aged 75 years or older – comparing the risk of complications with their younger counterparts
Source: Aging Clin Exp Res. 2024 May 8;36(1):107. doi: 10.1007/s40520-024-02751-5 (PMC11076407; doi:10.1007/s40520-024-02751-5)
Supplement: Supplementary file 1 — Supplementary Material 1 [file 40520_2024_2751_MOESM1_ESM.docx]

| Supplemental table 1. Logistic regression analysis on the association between age and postoperative complication adjusting for preoperative variables | | | | | | | | | | | | | | | | |
| --- | --- | --- | --- | --- | --- | --- | --- | --- | --- | --- | --- | --- | --- | --- | --- | --- |
| Univariate logistics regression | | | | | | | |  | Multivariate logistics regression - preoperative variables | | | | | | | |
|  |  |  | Any complication | |  | CD ≥III | |  |  |  |  | Any complication | |  | CD ≥III | |
|  |  |  | OR | 95% CI |  | OR | 95% CI |  |  |  |  | OR | 95% CI |  | OR | 95% CI |
| Age |  |  |  |  |  |  |  |  | Age |  |  |  |  |  |  |  |
|  | <55 |  | Ref |  |  | Ref |  |  |  | <55 |  | Ref |  |  | Ref |  |
|  | 55-64 |  | 1.70 | 0.95-3.09 |  | 4.74 | 1.21-31 |  |  | 55-64 |  | 1.55 | 0.82-3.00 |  | 3.49 | 0.80-24 |
|  | 65-74 |  | 1.57 | 0.90-2.81 |  | 5.67 | 1.54-37 |  |  | 65-74 |  | 1.35 | 0.69-2.65 |  | 3.78 | 0.88-26 |
|  | ≥75 |  | 2.15 | 1.07-4.31 |  | 5.05 | 1.05-36 |  |  | ≥75 |  | 1.82 | 0.80-4.13 |  | 3.21 | 0.57-25 |
| Sex |  |  |  |  |  |  |  |  | Gender |  |  |  |  |  |  |  |
|  | Female |  | Ref |  |  | Ref |  |  |  | Female |  | Ref |  |  | Ref |  |
|  | Male |  | 0.96 | 0.63-1.48 |  | 1.81 | 0.80-4.63 |  |  | Male |  | 0.91 | 0.57-1.45 |  | 1.69 | 0.69-4.60 |
| Charlson comorbidity index | |  |  |  |  |  |  |  | Charlson comorbidity index | | | | | | |  |
|  | 0 |  | Ref |  |  | Ref |  |  |  | 0 |  | Ref |  |  | Ref |  |
|  | 1 |  | 0.72 | 0.43-1.19 |  | 0.71 | 0.23-1.87 |  |  | 1 |  | 0.61 | 0.33-.11 |  | 0.31 | 0.08-1.00 |
|  | ≥2 |  | 2.09 | 1.18-3.67 |  | 3.07 | 1.28-7.08 |  |  | ≥2 |  | 1.47 | 0.71-3.01 |  | 0.87 | 0.26-2.77 |
| American Society of Anesthesiologists classification | | | | | | | |  | American Society of Anesthesiologists classification | | | | | | | |
|  | 1 |  | Ref |  |  | Ref |  |  |  | 1 |  | Ref |  |  | Ref |  |
|  | 2 |  | 1.43 | 0.78-2.77 |  | 1.16 | 0.36-5.16 |  |  | 2 |  | 1.58 | 0.79-3.35 |  | 1.43 | 0.35-9.62 |
|  | ≥3 |  | 1.89 | 0.97-3.83 |  | 3.08 | 0.97-13 |  |  | ≥3 |  | 1.86 | 0.76-4.67 |  | 3.40 | 0.65-26 |
| Body mass index | |  |  |  |  |  |  |  | Body mass index | |  |  |  |  |  |  |
|  | <25 |  | Ref |  |  | Ref |  |  |  | <25 |  | Ref |  |  | Ref |  |
|  | 25-29 |  | 0.67 | 0.40-1.12 |  | 0.55 | 0.20-1.37 |  |  | 25-29 |  | 0.69 | 0.40-1.18 |  | 0.59 | 0.21-1.54 |
|  | ≥30 |  | 0.81 | 0.49-1.34 |  | 0.57 | 0.21-1.42 |  |  | ≥30 |  | 0.95 | 0.55-1.63 |  | 0.72 | 0.25-1.99 |
| Smoking |  |  |  |  |  |  |  |  | Smoking |  |  |  |  |  |  |  |
|  | Never |  | Ref |  |  | Ref |  |  |  | Never |  | Ref |  |  | Ref |  |
|  | Active smoker |  | 0.97 | 0.56-1.67 |  | 1.65 | 0.59-4.63 |  |  | Active smoker |  | 0.93 | 0.50-1.71 |  | 1.68 | 0.53-5.35 |
|  | Former smoker |  | 1.54 | 0.96-2.48 |  | 2.24 | 0.94-5.70 |  |  | Former smoker |  | 1.42 | 0.85-2.38 |  | 1.90 | 0.71-5.45 |
| Tumor size |  |  |  |  |  |  |  |  | Tumor size |  |  |  |  |  |  |  |
|  | q1 |  | Ref |  |  | Ref |  |  |  | q1 |  | Ref |  |  | Ref |  |
|  | q2 |  | 1.49 | 0.88-2.56 |  | 1.13 | 0.43-3.99 |  |  | q2 |  | 1.59 | 0.90-2.82 |  | 1.44 | 0.45-5.03 |
|  | q3 |  | 0.86 | 0.41-1.72 |  | 1.38 | 0.34-4.99 |  |  | q3 |  | 0.85 | 0.39-1.77 |  | 1.49 | 0.34-6.21 |
|  | q4 |  | 1.82 | 1.05-3.19 |  | 2.86 | 1.09-8.38 |  |  | q4 |  | 1.54 | 0.84-2.84 |  | 2.52 | 0.83-8.69 |

Abbreviations: OR, odds ratio; CI, confidence interval; Ref, reference; q, quartile
