# Supplementary material for: Robot-assisted partial nephrectomy in patients aged 75 years or older – comparing the risk of complications with their younger counterparts
Source: Aging Clin Exp Res. 2024 May 8;36(1):107. doi: 10.1007/s40520-024-02751-5 (PMC11076407; doi:10.1007/s40520-024-02751-5)
Supplement: Supplementary file 2 — Supplementary Material 2 [file 40520_2024_2751_MOESM2_ESM.docx]

| Supplemental table 2. Logistic regression analysis on the association between age and postoperative complication adjusting for perioperative variables | | | | | | | | | | | | | | | | | |
| --- | --- | --- | --- | --- | --- | --- | --- | --- | --- | --- | --- | --- | --- | --- | --- | --- | --- |
| Univariate logistics regression | | | | | | | |  | Multivariate logistics regression - perioperative variables | | | | | | | | |
|  |  |  | Any complication | |  | CD ≥III | |  |  |  |  | Any complication | |  | | CD ≥III | |
|  |  |  | OR | 95% CI |  | OR | 95% CI |  |  |  |  | OR | 95% CI |  | | OR | 95% CI |
| Age |  |  |  |  |  |  |  |  | Age |  |  |  |  |  | |  |  |
|  | <55 |  | Ref |  |  | Ref |  |  |  | <55 |  | Ref |  |  | | Ref |  |
|  | 55-64 |  | 1.70 | 0.95-3.09 |  | 4.74 | 1.21-31 |  |  | 55-64 |  | 1.29 | 0.64-2.63 |  | | 3.20 | 0.75-22 |
|  | 65-74 |  | 1.57 | 0.90-2.81 |  | 5.67 | 1.54-37 |  |  | 65-74 |  | 1.30 | 0.65-2.65 |  | | 2.67 | 0.62-18 |
|  | ≥75 |  | 2.15 | 1.07-4.31 |  | 5.05 | 1.05-36 |  |  | ≥75 |  | 1.98 | 0.86-4.58 |  | | 2.48 | 0.43-19 |
| Duration procedure | |  |  |  |  |  |  |  | Duration procedure | |  |  |  |  |  | |  |
|  | q1 |  | Ref |  |  | Ref |  |  |  | q1 |  | Ref |  |  | | Ref |  |
|  | q2 |  | 1.67 | 0.84-3.36 |  | 2.64 | 0.55-18 |  |  | q2 |  | 1.47 | 0.71-3.08 |  | | 2.64 | 0.53-19 |
|  | q3 |  | 1.39 | 0.69-2.83 |  | 2.62 | 0.55-18 |  |  | q3 |  | 0.92 | 0.42-1.99 |  | | 1.70 | 0.30-13 |
|  | q4 |  | 3.00 | 1.56-5.93 |  | 7.60 | 2.02-49 |  |  | q4 |  | 1.69 | 0.80-3.60 |  | | 4.33 | 1.02-30 |
| Warm ischemia time | |  |  |  |  |  |  |  | Warm ischemia time | |  |  |  |  |  | |  |
|  | q1 |  | Ref |  |  | Ref |  |  |  | q1 |  | Ref |  |  | | Ref |  |
|  | q2 |  | 1.78 | 0.98-3.26 |  | 2.44 | 0.75-9.34 |  |  | q2 |  | 1.64 | 0.78-3.48 |  | | 0.84 | 0.18-3.88 |
|  | q3 |  | 1.70 | 0.94-3.10 |  | 2.59 | 0.82-9.79 |  |  | q3 |  | 1.71 | 0.80-3.71 |  | | 1.18 | 0.30-5.13 |
|  | q4 |  | 2.55 | 1.41-4.70 |  | 3.17 | 0.99-12 |  |  | q4 |  | 2.28 | 1.09-4.87 |  | | 1.31 | 0.36-5.44 |
| Blood loss |  |  |  |  |  |  |  |  | Blood loss |  |  |  |  |  | |  |  |
|  | q1 |  | Ref |  |  | Ref |  |  |  | q1 |  | Ref |  |  | | Ref |  |
|  | q2 |  | 0.81 | 0.41-1.52 |  | 3.47 | 0.83-17 |  |  | q2 |  | 0.71 | 0.31-1.52 |  | | 1.09 | 0.13-7.13 |
|  | q3 |  | 2.42 | 1.35-4.35 |  | 6.89 | 1.93-32 |  |  | q3 |  | 1.89 | 0.94-3.81 |  | | 4.52 | 1.15-22 |
|  | q4 |  | 2.94 | 1.85-5.01 |  | 9.61 | 3.07-42 |  |  | q4 |  | 2.16 | 1.11-4.21 |  | | 3.96 | 1.06-19 |

Abbreviations: OR, odds ratio; CI, confidence interval; Ref, reference; q, quartile.
